# Supplementary figures and images for: DMSO induces major morphological and physiological alterations in zebrafish embryos
Source: PLoS One. 2025 Aug 18;20(8):e0330348. doi: 10.1371/journal.pone.0330348 (PMC12360551; doi:10.1371/journal.pone.0330348)

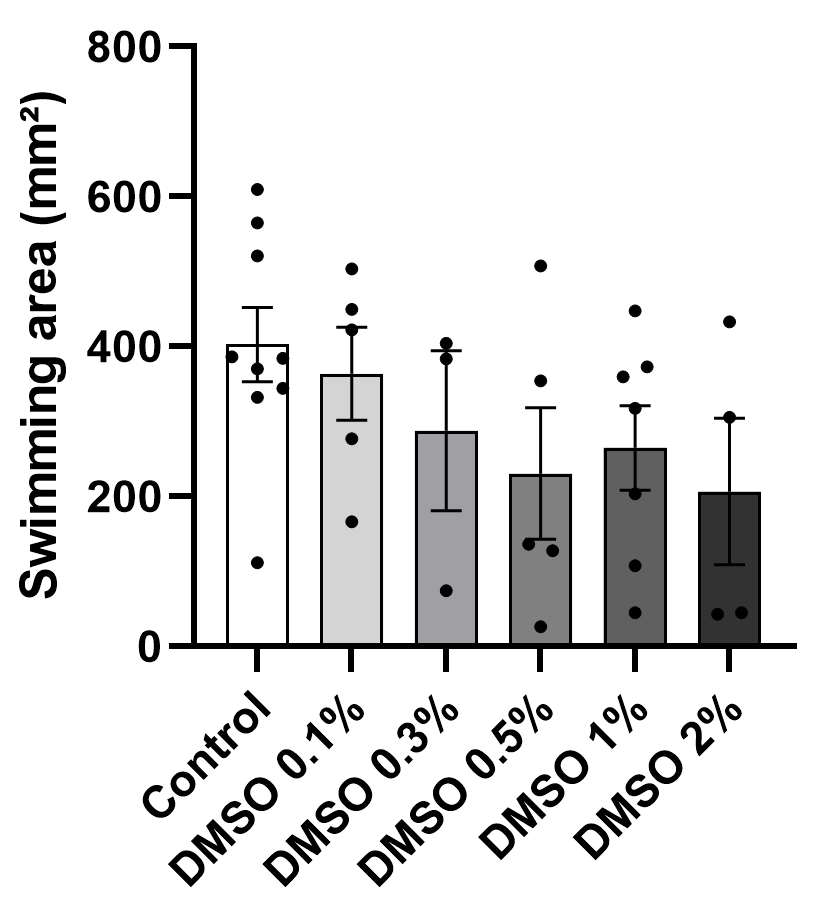

Supplement: S3 File — Larvae with 7 dpf were placed in 35-mm culture dishes filled with 2 mL of E3 solution. Movement of control and DMSO-treated embryos were recorded with a cell phone for 10 minutes. Eight independent experiments were performed with control, 0.1% DMSO, 0.3% DMSO, 0.5% DMSO, 1% DMSO and 2% DMSO. No significant alterations in embryos motility were observed, although a dose-dependent tendency of decrease in locomotion was observed. Number of embryos analyzed per experimental group = 80. (TIF) [file pone.0330348.s004.tif]
